# ECMO in neonates: The association between cerebral hemodynamics with neurological function

**DOI:** 10.3389/fped.2022.908861

**Published:** 2022-09-06

**Authors:** Shu-Han Yu, Dan-Hua Mao, Rong Ju, Yi-Yong Fu, Li-Bing Zhang, Guang Yue

**Affiliations:** ^1^Department of Neonatology, Chengdu Women's and Children's Central Hospital, School of Medicine, University of Electronic Science and Technology of China, Chengdu, China; ^2^Department of Pediatric Surgery, Chengdu Women's and Children's Central Hospital, School of Medicine, University of Electronic Science and Technology of China, Chengdu, China

**Keywords:** extracorporeal membrane oxygenation, neonate, hemodynamics, neurologic function, blood vessel

## Abstract

Extracorporeal membrane oxygenation (ECMO) is a superior life support technology, commonly employed in critical patients with severe respiratory or hemodynamic failure to provide effective respiratory and circulatory support, which is especially recommended for the treatment of critical neonates. However, the vascular management of neonates with veno-arterial extracorporeal membrane oxygenation (VA-ECMO) is still under controversy. Reconstruction or ligation for the right common carotid artery (RCCA) after ECMO is inconclusive. This review summarized the existed studies on hemodynamics and neurological function after vascular ligation or reconstruction hoping to provide better strategies for vessel management in newborns after ECMO. After reconstruction, the right cerebral blood flow can increase immediately, and the normal blood supply can be restored rapidly. But the reconstructed vessel may be occluded and stenotic in long-term follow-ups. Ligation may cause lateralization damage, but there could be no significant effect owing to the establishment of collateral circulation. The completion of the circle of Willis, the congenital anomalies of cerebral or cervical vasculature, the duration of ECMO, and the vascular condition at the site of arterial catheterization should be assessed carefully before making the decision. It is also necessary to follow up on the reconstructed vessel sustainability, and the association between cerebral hemodynamics and neurological function requires further large-scale multi-center studies.

## Introduction

Extracorporeal membrane oxygenation (ECMO) can reduce the mortality of neonates with reversible cardiopulmonary failure, which is difficult to treat with conventional mechanical ventilation (1, 2), approximately from 80 to 20%. The outcomes of neonates with critical illnesses such as congenital diaphragmatic hernia, persistent pulmonary hypertension, respiratory distress syndrome, or congenital heart diseases in NICU have improved significantly over the decades since the advent of ECMO (3, 4). Except for the central cannulation draining from the right atrium returning into the aortic arch after chest opening (5, 6), the common way to establish neonatal arteriovenous access is to connect the proximal end of the corresponding vessels to ECMO machines, followed by ligating the distal end of the right common carotid artery (RCCA) and the right jugular vein (7, 8). In terms of the distal vessel management, some studies recommend reconstructing the RCCA because of the occurrence of a right hemisphere injury after ligation (9), and due to the rapidly-increasing right cerebral blood flow which improves the brain tissue supply after reconstruction. However, reconstruction is not recommended by other studies, due to the risk of stenosis and occlusion occurring in the reconstructed vasculature, which may affect the blood supply in long-term follow-ups (10). Moreover, some studies showed that after ligation, the immediate establishment of collateral circulation was sufficient to maintain the blood supply to the right cerebral hemisphere. In addition, no lateralization injury was found in either of the cerebral hemispheres (11, 12). Therefore, it is still under debate whether to apply ligation or reconstruction in neonates who had ECMO (13). Thus, numerous studies have focused on the association between the cerebral hemodynamic patterns and neurological function in neonates with ECMO after arteriovenous catheterization.

## Hemodynamics during ECMO

ECMO can be divided into two modes in terms of the blood return: veno-venous ECMO (VV-ECMO) and veno-arterial ECMO (VA-ECMO). Arterial drainage was adopted in the VA-ECMO mode. For adults and children, multiple arteries could be selected to establish circulation, and the artery could be repaired immediately instead of permanent ligation. However, for newborns, the selections are limited due to the tenuous blood vessel wall. The RCCA is the most commonly used vessel for catheterization and ligation, especially for premature babies. Thus, the blood supply to the right hemisphere is completely cut off. ECMO should be started immediately after catheterization to restore the blood supply to the right brain through collateral circulation. Cephalic vascular ultrasound is an important measurement for monitoring the cerebral blood supply during ECMO. In general, the blood flow velocity of major cerebral arteries in the systolic (Vs) and diastolic (Vd) stages reflects the changes in the cerebral blood flow. Abnormal transcranial Doppler flow velocity may precede neurological injury in children with ECMO, and these abnormalities include intracranial hemorrhage, increased intracranial pressure, and ischemic injury (14). Therefore, the evaluation of the blood flow velocity in the major brain vessels is crucial for neurological prognosis. The middle cerebral artery (MCA) had an obvious decrease in the peak systolic velocity (PSV) after ligation, which increased back to 70% of baseline within 3 to 5 min. Conversely, the end diastolic velocity (EDV) remained unchanged initially, and increased from baseline within 3 to 5 min. The flow of the MCA was anterograde throughout the ECMO operation, showing that collateral circulation was established instantaneously after ligation and increased in a short time (15), consistent with the changes in cerebral oxygenation measured by near-infrared spectrophotometry (NIRS) after the initiation of ECMO and the ligation of the RCCA (16).

Hemodynamic changes occurred in carotid arteries while ECMO was initiated: The velocity of the blood flow in the proximal right internal carotid artery showed a significant decrease, the PSV dropped sharply, and the EDV remained basically unchanged. However, the blood flow elevated on the other side suggesting that the bilateral intracranial blood supply was restored by a compensatory increase in the left common carotid artery (15, 17). In addition, the right external carotid artery and ophthalmic artery may also provide collateral blood flow by anastomosis with the left carotid artery (7, 17). Correspondingly, intracranial collateral pathways were established through the circle of Willis, including flow from left to right in the anterior communicating artery and retrograde flow from the right anterior cerebral artery A1 segment. Hence, the compensatory blood flow was increased in the left brain maintaining the blood supply for both hemispheres. Arno et al. found that the average velocity of the left MCA was elevated and that reflux flow appeared in the right internal carotid artery in some newborns, even in the subclavian artery steal (18). However, the correlation between such changes in the internal carotid blood flow and direction with brain injury were still undefined (17, 19, 20). The MCA flow increased in patients with intracerebral hemorrhage in one study (14); in contrast, changes in the cerebral artery flow velocity showed no correlation with acute nerve injuries (ANI) such as intracranial hemorrhage, ischemic injury, or epilepsy in other studies (21, 22). However, they all agreed that the increased pulsatility index of arteries might be a marker of a cerebral ischemia injury (14, 22). Jay et al. suggested that change in a single arterial flow was not significantly associated with ANI (17). The flow velocity of the right posterior cerebral artery was significantly higher than that of the left and the systolic flow of the left middle cerebral artery was higher than that of the right after ECMO initiation, which may indicate a neurological injury throughout the disturbance and asymmetrical distribution of blood flow in different cerebral arteries, as hemorrhagic lesions tend to occur in the left brain while ischemic injury increases in the right (9, 23). Contrary to Arno's study, there was no different impact on the left and right hemispheres due to changes in hemodynamics (18).

Apart from the changes in arteries, it was found that the changes in veins caused by ligation might be related to brain injury. There might be a relationship between the continuous decrease in the blood flow velocity of the superior sagittal sinus after ligating the right jugular vein and cerebrovascular injury in neonates (24). It was found that a significant decrease in the left MCA flow following the close of the cephalic venous drainage and a considerable increase in the resistance index. This change disappeared after cephalic vein drainage opening, which suggested that the cephalic venous drainage could maintain normal cerebral blood flow and might reduce the possibility of brain injury (25). However, some researchers hold the opposite view that this injury may not be caused by abnormal venous drainage, since approximately half of the patients showed no change in the flow velocity in the superior sagittal sinus after venous drainage close; meanwhile, there was evidence that the drainage patterns varied from entirely one single jugular vein to both jugular veins and vertebral veins after a brief occlusion of one or both jugular veins in a healthy newborn, and this met the demand of adequate cerebral venous drainage (26). Therefore, it seemed that the hemodynamic changes in the intracranial venous blood flow during ECMO were unlikely to be due to partial obstruction of cephalic venous drainage caused by intravenous catheterization.

ANI was more strongly associated with the changes in the cerebral arterial blood flow than veins, which had more collateral circulation and were also related to brain injury by affecting the hemodynamics of the cerebral arteries. During the running of ECMO, most studies hold that the interruption of the cerebral blood flow could be restored in a short time by a complete circle of Willis and collateral circulations. The asymmetric flow pattern after ECMO initiation could potentially cause ANI, and the RI might be an important predictor. Additionally, autoregulatory functions achieved by cerebral vasoconstriction and relaxation may be impaired in neonates in various severe disease states (27). With the common involvement of the two above, both hypoperfused and hyperperfused blood flow states occurring in cerebral hemispheres may contribute to severe acute neurological damage and even play an important role in long-term neurological dysfunction.

## Hemodynamics after ECMO

### Hemodynamics after ligation

During medium and long-term follow-ups, the blood flow velocity and vessel diameter of the right carotid artery were smaller than those of the left carotid artery in patients with permanent ligation of the RCCA (28). After ligation, the flow velocity of the right anterior cerebral artery A1 segment decreased, yet increased in the left anterior cerebral artery, and the flow velocity of the right posterior cerebral artery was significantly higher than that of the left. This abnormal increase in the cerebral blood flow may be related to brain injury (15, 17). At present, there is no definite evidence for long-term neurological deficits after permanent ligation, but it remains unknown whether such compensatory cerebral perfusion meets the needs of neonatal brain development.

### Hemodynamics after reconstruction

For patients with the RCCA reconstruction, the flow velocities of the right internal carotid artery and bilateral anterior and middle cerebral artery were generally high, and the distribution became more symmetrical which increased the blood flow of the left and right MCA immediately after extubation (7, 15). The blood flow direction and velocity of the left and right carotid arteries after reconstruction were the same as those of normal neonates in the same age (19). Another study using SPECT also showed that the cerebral blood flow was restored to normal after reconstruction (29). Moulton et al. (30) found that the mean blood flow velocity of the right internal carotid artery was slightly slower than that of the left within 24 h after reconstruction, but gradually recovered and was even faster than the left artery within 4 to 7 months after reconstruction. In summary, there is no doubt that blood flow will be restored in the cerebral hemispheres after reconstruction. However, it is not clear whether it could reverse the potential neurological injury caused by the short-term insufficient blood supply to the right cerebral hemisphere during ECMO. In addition, stenosis or occlusion of reconstructed vessels and reperfusion injury could affect the long-term outcome of reconstruction, and may even cause additional neurological impairment (31).

### Vascular patency after reconstruction

The patency of the reconstructed RCCA may compromise the restoration of the right cerebral blood supply, and associated vascular complications may occur. More than half of the reconstructed RCCAs were either occlusive or severely stenotic in a long-term MRA follow-up study, but almost all of the right internal carotid arteries were patent, which suggested that there was an extracranial collateral blood supply, despite the shrunken internal carotid artery (10). A 4-year follow-up study that evaluated children with the RCCA reconstruction found that the reconstructed artery had no structural or hemodynamic impairments, and the patency rate was the highest in the neonatal period but gradually decreased over time (32). The right subcortical cerebral blood flow was decreased when the reconstructed RCCA was obstructed, but the bilateral subcortical cerebral blood flow was still higher than that in the other critically ill children without ECMO. The brain perfusion could be maintained by the anterior and posterior communicating arteries or extracranial collateral vessels despite stenosis or occlusion after the RCCA reconstruction. The extracranial collateral vessels were mainly filled by the right internal carotid artery with the thyrocervical trunk, the right external carotid artery and the occipital artery (33), which may be similar to permanent ligation of the RCCA.

The others hold the oppositive view that most of the reconstructed RCCAs were patent and only a small part were stenotic or occluded (7, 8, 15). A recent study of infants except for newborns showed that approximately half of the RCCAs were patent after repair and could be used for intubation to perform ECMO again (13). Moreover, the duration of ECMO was shorter in patients whose RCCA was patent after repair than in those whose RCCA was not (34), which may be associated with less injury in the arterial intima caused by a shorter duration of ECMO (35). Tissue biopsies have suggested that circular transmural necrosis (CTN) mainly characterized by vessel wall necrosis and elastic fiber fragmentation was common surrounding the catheterization site, and it was correlated with the range of CTN and the duration of ECMO. In addition, limited subintimal necrosis or localized transmural necrosis was also present at the proximal part of the catheterization site (30).

Levy et al. (36) conducted a study to assess the degree of stenosis for the RCCA reconstruction by using the diameter index (DI, the diameter of the anastomosis / the diameter of 5 mm proximal to the anastomosis), which was determined at the 1 week, 6 to 9 months, and 4 years follow-up visits, and the RCCA reconstruction had a high patency rate and no relevant manifestations such as hemangioma after reconstruction. However, it was also shown that stenosis of the RCCA was more common in the early stage, and the DI tended to increase with the gradually alleviative stenosis over time. The higher patency rate may be associated with the strict criteria used in this research: reconstruction was not performed when there was dissection of the proximal or distal intimal, arterial dissection, arterial thrombus, disappearance of free flow, or high tension at the anastomotic site. These differences in the patency studies may be related to the manners of reconstruction and vascular circumstances. Thus, the vitality of surrounding vessels and tissue should be evaluated before reconstruction, and the patency after reconstruction and its variation need to be further followed-up over time.

Different management protocols of the RCCA after weaning produce different hemodynamic changes: after ligation, there was a compensatory increase in the left cerebral blood flow, which provided compensation to the right hemisphere through the circle of Willis. Whether this pattern of blood flow can meet the needs of the neonate's neurodevelopment also requires long-term neurological studies. The immediate and long-term studies that evaluated the results after the RCCA reconstruction showed restoration of blood flow in the right cerebral hemisphere. Therefore, the authors believed that the RCCA reconstruction should be performed aggressively to restore the normal physiological structures whenever possible. However, the reconstruction was affected by many aspects, such as the duration of ECMO and the viability of the surrounding vascular tissue. Hence, a comprehensive evaluation was needed. Also, whether reconstruction can reverse the short-term ischemic injury during ECMO and the reperfusion injury after restoration still lacks corresponding clinical research evidence. Studies have shown that the patency rate of the reconstructed RCCA decreases gradually with increasing age. Because a lack of compensatory mechanisms, stenosis, and occlusion of carotid arteries had a huge impact on the adult cerebral blood supply and systemic blood circulation (37, 38), long-term and sustained follow-up of the reconstructed RCCA patency is highly necessary.

## Evaluation of the nervous system after reconstruction or ligation

### Assessment of imaging

Hemorrhagic or ischemic infarcts and diffuse atrophy were typically included in the abnormal imaging findings in children after ECMO. Some studies hold that these findings showed no difference between the two cerebral hemispheres in children with the RCCA ligation (8) and were not correlated with the arterial flow velocity in the left and right cerebral hemisphere (19). Cranial CT or MRI also showed no difference in the long-term neurological outcomes between ligation and reconstruction (15, 35), and brain CT performed before and after anastomotic stricture in children with the reconstructed RCCA also showed the same result (32). These research studies concluded that there was no difference in the neurological injuries caused by ligation or reconstruction in either hemisphere. In contrast, MRA examination of children with ECMO suggested that focal cerebral lesions might be related to the asymmetric blood flow pattern of the right and left MCA after ligation (39). Therefore, it is possible that artery ligation may lead to a unilateral brain injury. Moreover, some studies found no lateralization abnormalities of the bilateral cerebral hemispheres in children after the RCCA reconstruction (10, 19, 36). Considering this opinion, it seems that reconstruction is beneficial to eliminate the lateralization injury.

### Assessment of neurofunction

Electroencephalogram (EEG) is an important tool for neurological assessment. Early monitoring of EEG is necessary during ECMO, especially after ECMO initiation to 48 h after extubation, when the degree of abnormalities and the severity of the neuroimaging findings are clearly correlated and seem to predict the neurological outcome (40, 41). Electroconvulsive features were reported to be found on the EEG of the ipsilateral side of children on ECMO with unilateral arterial cannulation, which seemed to illustrate the association of abnormal cerebral hemodynamic changes and impairments of neurological function (42). In a retrospective study of 59 neonates who survived after ECMO, Schumacher et al. (9) found that eight patients had varying degrees of injury within the right hemisphere and the EEG showed an increased incidence of background slowing, attenuation, and diffuse abnormalities in the right hemisphere (9). In infants, cerebral palsy was more likely to occur after ligation instead of reconstruction (8), and focal convulsion was much more common on the right side (23). For infants with the RCCA ligation, neurophysiological abnormalities of the right hemisphere seemed to be more common than those of the left hemisphere. But some researchers found that the EEGs of the reconstructed and ligated groups were basically the same on both sides (43).

The auditory threshold, auditory evoked potential latency, V wave amplitude of automatic auditory brainstem response, auditory P30 wave of evoked potential, and N12 wave amplitude of somatosensory evoked potential all showed no significant difference in long-term follow-up of the brain bilaterally (28). In neonates, brain metabology studies have suggested a decrease in N-acetyl-aspartate and an increase in lactic acid, which were associated with adverse neurodevelopmental outcomes. However, there was no difference in the levels of N-acetyl-aspartate and lactic acid in the bilateral basal ganglia by 1H-MRS examination after ligation (44, 45).

In terms of the long-term neurological assessment, intellectual impairment, hearing impairment, and cerebral palsy were the common sequelae in children after ECMO. No significant difference was found in the intelligence quotient between the reconstruction and ligation group (8, 46). Long-term neuropsychological deficits were associated with the underlying disease processes in the neonatal period instead of with ECMO treatment (47). In contrast, some studies found that the duration of ECMO treatment was associated with ANI in children, while the long-term neurological prognosis was favorable (48). At present, there is no definite evidence that either reconstruction or ligation will lead to long-term neurological abnormalities. More prospective comparative studies are urgently needed.

### Assessment of cerebral oxygenation

In recent years, NIRS has provided a new perspective estimating the neurological outcomes by monitoring the cerebral oxygen saturation (rScO2). The rScO2 decreased by 12 to 25% from the baseline in the right frontal region after ligation and this lasted for a few minutes, and then returned to the baseline level, while there was no obvious change in the left. Subsequently, transient elevation of rScO2 was observed in both cerebral hemispheres upon the initiation of ECMO (16). In addition, a study that monitored the cerebral oxygenation and related indicators by the NIRS and that explored the relationship with cerebral autoregulatory function, found that the brain autoregulatory function might be impaired when the blood flow was lower during ECMO (49). The right cerebral blood flow decreased during ECMO and was more susceptible to the interference of autoregulatory function, which seemed to explain the susceptibility to ANI in the right cerebral hemisphere. Additional studies showed that decreased cerebral oxygenation was associated with adverse short-term neurological outcomes and death (50–53). In addition, a high rScO2 (>80%) was a protective factor that might predict a lower in-hospital mortality (54). Therefore, cerebral oxygen monitoring may serve as a potential important method for monitoring the neurological prognosis and clinical outcomes in neonates with ECMO. Further studies focusing on cerebral oxygen saturation after the RCCA ligation and reconstruction may help to assess whether to reconstruct or not.

Non-invasive monitoring methods of neurological function were feasible in neonatal ECMO, such as EEG, somatosensory evoked potentials (SSEP) and NIRS (55). Convulsive seizures, in particular, were often suggestive of moderate to severe brain damage and were correlated strongly with the neuroimaging findings. Although studies have found that neonates with ECMO have more neurological impairment than those without. But there is still considerable heterogeneity in the available studies in terms of the long-term neurological outcomes, and there is currently no clear evidence pointing to the superiority of either reconstruction or ligation (56–59). It is imperative to provide more definitive clinical decisions on vascular management by a standardized follow-up program (60), and more prospective studies are needed to evaluate the correlation between cerebral hemodynamics and neurological function.

## Effect of anatomical variations on reconstruction and ligation

The circle of Willis is an important anatomic structure to guarantee the blood supply to the right brain in children with ECMO, and there is a large variability in the general population (35). The congenital anatomic variations of the circle of Willis may have an important impact on the hemodynamic and vascular changes after ligation. Reconstruction may be beneficial in children with an incomplete circle of Willis after ECMO (61). In addition, in children with a common origin of the carotid arteries (COCA), the risk of neurological injury did not increase after ligation, but further evaluation is needed (62).

The completeness of the circle of Willis and congenital variations of the cerebral and cervical vasculature may have a profound impact on the choice of reconstruction or permanent ligation after ECMO.

## Management of vascular complications after reconstruction

During ECMO, the application of anticoagulant therapy is necessary, because the damage to the vessel wall caused by intubation leads to the occurrence of cascades of coagulation and inflammation (63, 64). Additionally, the contact of the blood with the non-vessel walls in the machine line, also stimulate the coagulation cascade (65). During ECMO, the anticoagulation strategies should be more aggressively and dynamically adjusted due to the presence of “developmental hemostasis” (66, 67). Previous studies showed a 15 to 85% incidence of catheter-related deep vein thrombosis after extubation in adult patients who had the VV ECMO (68–70). In addition, some patients developed arterial complications such as limb ischemia and arterial stenosis (70). For neonatal patients, reconstruction with the RCCA may be of great importance to maintain the normal physiological status when possible, as described previously. But the formation of arteriovenous thrombi and stenoses can make a profound hemodynamic impact on the brain function. It should be monitored more closely for vascular morphology and function after reconstruction. Currently, there is no common view on the use of anticoagulants after weaning. Some research studies have suggested the routine use of duplex ultrasound for follow-up of vascular complications after extubation in adult patients; anticoagulation therapy should be continued for at least 3 months in adult patients who developed catheter-related deep vein thrombosis, and follow-up should be continued until the thrombus disappears (68, 71). However, for neonatal patients, the related clinical data are very scarce, and further clinical studies are needed to evaluate the type of anticoagulant drugs and the timing of their use after weaning. Additionally, whether the use of anticoagulant drugs is meaningful for maintaining the patency of the reconstructed vessels also needs further study.

There may be vascular complications after ECMO, especially for the reconstructed vessels, and a long-term follow-up of the vascular function and morphology is important. Anticoagulation medication after weaning is still controversial, and the treatment should be dynamically adjusted according to the vascular findings on follow-up. Further clinical studies should be performed.

## Conclusion

Reconstruction or ligation for the RCCA after ECMO is still controversial. The completion of the circle of Willis, the congenital anomalies of cerebral or cervical vasculature, the duration of ECMO and the vascular condition at the site of arterial catheterization should be assessed carefully before making the decision. It is also necessary to follow up on the reconstructed vessel sustainability, and the association between cerebral hemodynamics and neurological function requires further large-scale multi-center studies.

**Figure 1:**
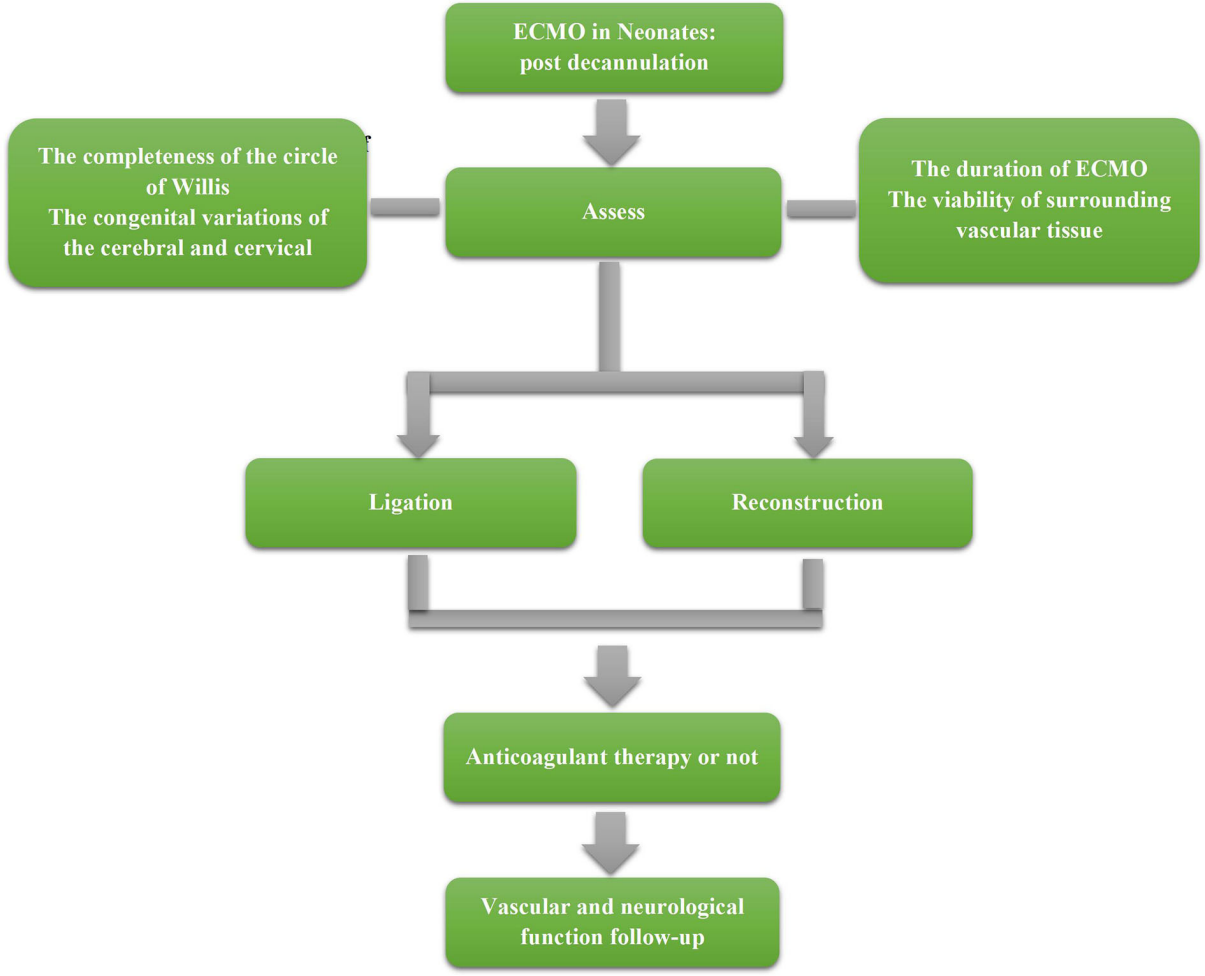
The flow chart of vascular image management post decannulation.

A suggested management strategy post decannulation is shown in Figure 1. However, limited to the existing studies, the content in management still cannot be clearly stated. Therefore, this flow chart can only reflect the preliminary management strategy, and more studies are needed to refine the management process.

## Author contributions

S-HY and D-HM contributed to the study conception and design, collection, analysis and interpretation of data, and drafting of the manuscript. GY contributed to study conception and design, critical revision of the manuscript for the important intellectual contents, and final approval of the manuscript. RJ and Y-YF contributed to the data collection and critical revision of the manuscript for the important intellectual contents. L-BZ contributed to the critical revision of the manuscript for the important intellectual contents. All authors approved the final manuscript as submitted and agree to be accountable for all aspects of the work.

## Conflict of interest

The authors declare that the research was conducted in the absence of any commercial or financial relationships that could be construed as a potential conflict of interest.

## Publisher's note

All claims expressed in this article are solely those of the authors and do not necessarily represent those of their affiliated organizations, or those of the publisher, the editors and the reviewers. Any product that may be evaluated in this article, or claim that may be made by its manufacturer, is not guaranteed or endorsed by the publisher.

## References

[1] Bartlett RH Roloff DW Cornell RG Andrews AF Dillon PW Zwischenberger JB. Extracorporeal circulation in neonatal respiratory failure: a prospective randomized study. Pediatrics. (1985) 76:479–87. 10.1542/peds.76.4.479 3900904

[2] O'Rourke PP Crone RK Vacanti JP Ware JH Lillehei CW Parad RB. Extracorporeal membrane oxygenation and conventional medical therapy in neonates with persistent pulmonary hypertension of the newborn: a prospective randomized study. Pediatrics. (1989) 84:957–63. 10.1542/peds.84.6.957 2685740

[3] Grover TR Rintoul NE Hedrick HL. Extracorporeal membrane oxygenation in infants with congenital diaphragmatic hernia. Semin Perinatol. (2018) 42:96–103. 10.1053/j.semperi.2017.12.005 29338874

[4] Hong X Zhao Z Liu Z Liu C Wang J Quan X. Venoarterial extracorporeal membrane oxygenation for severe neonatal acute respiratory distress syndrome in a developing country. Front Pediatr. (2020) 8:227. 10.3389/fped.2020.00227 32548079 PMC7270410

[5] Roeleveld PP Mendonca M. Neonatal cardiac ECMO in 2019 and beyond. Front Pediatr. (2019) 7:327. 10.3389/fped.2019.00327 31497583 PMC6712998

[6] Harvey C. Cannulation for neonatal and pediatric extracorporeal membrane oxygenation for cardiac support. Front Pediatr. (2018) 6:17. 10.3389/fped.2018.00017 29616201 PMC5868139

[7] Graziani LJ Gringlas M Baumgart S. Cerebrovascular complications and neurodevelopmental sequelae of neonatal ECMO. Clin Perinatol. (1997) 24:655–75. 10.1016/S0095-5108(18)30163-5 9394865

[8] McCutcheon KC Wise L Lewis K Gilbert B Bhatia J Stansfield BK. The utility of cranial ultrasound as a screening tool for neonatal ECMO. J Perinat Med. (2020) 48:173–8. 10.1515/jpm-2019-0234 31821168

[9] Schumacher RE Barks JD Johnston MV Donn SM Scher MS Roloff DW. Right-sided brain lesions in infants following extracorporeal membrane oxygenation. Pediatrics. (1988) 82:155–61. 10.1542/peds.82.2.155 3399288

[10] Buesing KA Kilian AK Schaible T Loff S Sumargo S Neff KW. Extracorporeal membrane oxygenation in infants with congenital diaphragmatic hernia: follow-up MRI evaluating carotid artery reocclusion and neurologic outcome. AJR Am J Roentgenol. (2007) 188:1636–42. 10.2214/AJR.06.1319 17515387

[11] Perlman JM Altman DI. Symmetric cerebral blood flow in newborns who have undergone successful extracorporeal membrane oxygenation. Pediatrics. (1992) 89:235–9. 1734389

[12] Taylor GA Fitz CR Glass P Short BL. CT of cerebrovascular injury after neonatal extracorporeal membrane oxygenation: implications for neurodevelopmental outcome. AJR Am J Roentgenol. (1989) 153:121–6. 10.2214/ajr.153.1.121 2660530

[13] Carpenter JL Baker M Sperberg K Berger JT Vezina G Sinha P. Common carotid artery imaging after vessel sparing decannulation from Extracorporeal Membrane Oxygenation (ECMO) support. J Pediatr Surg. (2021) 56:2305–10. 10.1016/j.jpedsurg.2021.01.048 33632514

[14] O'Brien NF Hall MW. Extracorporeal membrane oxygenation and cerebral blood flow velocity in children. Pediatr Crit Care Med. (2013) 14:e126–34. 10.1097/PCC.0b013e3182712d62 23392359 PMC3873647

[15] Matsumoto JS Babcock DS Brody AS Weiss RG Ryckman FG Hiyama D. Right common carotid artery ligation for extracorporeal membrane oxygenation: cerebral blood flow velocity measurement with Doppler duplex US. Radiology. (1990) 175:757–60. 10.1148/radiology.175.3.2188299 2188299

[16] Ejike JC Schenkman KA Seidel K Ramamoorthy C Roberts JS. Cerebral oxygenation in neonatal and pediatric patients during veno-arterial extracorporeal life support. Pediatr Crit Care Med. (2006) 7:154–8. 10.1097/01.PCC.0000200969.65438.83 16446597

[17] Rilinger JF Smith CM. deRegnier RAO, Goldstein JL, Mills MG, Reynolds M, et al. Transcranial Doppler identification of neurologic injury during pediatric extracorporeal membrane oxygenation therapy. J Stroke Cerebrovasc Dis. (2017) 26:2336–45. 10.1016/j.jstrokecerebrovasdis.2017.05.022 28583819

[18] Van Heijst A Liem D Hopman J Van Der Staak F Sengers R. Oxygenation and hemodynamics in left and right cerebral hemispheres during induction of veno-arterial extracorporeal membrane oxygenation. J Pediatr. (2004) 144:223–8. 10.1016/j.jpeds.2003.11.006 14760266

[19] Lohrer RM Bejar RF Simko AJ Moulton SL Cornish JD. Internal carotid artery blood flow velocities before, during, and after extracorporeal membrane oxygenation. Am J Dis Child. (1992) 146:201–7. 10.1001/archpedi.1992.02160140067024 1733151

[20] Lazar EL Abramson SJ Weinstein S Stolar CJ. Neuroimaging of brain injury in neonates treated with extracorporeal membrane oxygenation: lessons learned from serial examinations. J Pediatr Surg. (1994) 29:186–91. 10.1016/0022-3468(94)90315-8 8176589

[21] Taylor GA Short BL Glass P Ichord R. Cerebral hemodynamics in infants undergoing extracorporeal membrane oxygenation: further observations. Radiology. (1988) 168:163–7. 10.1148/radiology.168.1.3289088 3289088

[22] O'Brien NF Buttram SDW Maa T Lovett ME Reuter-Rice K LaRovere KL. Cerebrovascular physiology during pediatric extracorporeal membrane oxygenation: a multicenter study using transcranial Doppler ultrasonography. Pediatr Crit Care Med. (2019) 20:178–86. 10.1097/PCC.0000000000001778 30395027

[23] Campbell LR Bunyapen C Holmes GL Howell CG Jr Kanto WP Jr. Right common carotid artery ligation in extracorporeal membrane oxygenation. J Pediatr. (1988) 113:110–3. 10.1016/S0022-3476(88)80543-2 3385518

[24] Taylor GA Walker LK. Intracranial venous system in newborns treated with extracorporeal membrane oxygenation: Doppler US evaluation after ligation of the right jugular vein. Radiology. (1992) 183:453–6. 10.1148/radiology.183.2.1561349 1561349

[25] Weber TR Kountzman B. The effects of venous occlusion on cerebral blood flow characteristics during ECMO. J Pediatr Surg. (1996) 31:1124–7. 10.1016/S0022-3468(96)90100-1 8863247

[26] Cowan F Thoresen M. Ultrasound study of the cranial venous system in the human new-born infant and the adult. Acta Physiol Scand. (1983) 117:131–7. 10.1111/j.1748-1716.1983.tb07187.x 6858701

[27] Rhee CJ da Costa CS Austin T Brady KM Czosnyka M Lee JK. Neonatal cerebrovascular autoregulation. Pediatr Res. (2018) 84:602–10. 10.1038/s41390-018-0141-6 30196311 PMC6422675

[28] Lott IT McPherson D Towne B Johnson D Starr A. Long-Term neurophysiologic outcome after neonatal extracorporeal membrane oxygenation. J Pediatr. (1990) 116:343–9. 10.1016/S0022-3476(05)82818-5 2407817

[29] Park CH Spitzer AR Desai HJ Zhang JJ Graziani LJ. Brain SPECT in neonates following extracorporeal membrane oxygenation: evaluation of technique and preliminary results. J Nucl Med. (1992) 33:1943–8. 1432154

[30] Moulton SL Lynch FP Cornish JD Bejar RF Simko AJ Krous HF. Carotid artery reconstruction following neonatal extracorporeal membrane oxygenation. J Pediatr Surg. (1991) 26:794–9. 10.1016/0022-3468(91)90141-F 1895187

[31] Young TL Quinn GE Baumgart S Petersen RA Schaffer DB. Extracorporeal membrane oxygenation causing asymmetric vasculopathy in neonatal infants. J AAPOS. (1997) 1:235–40. 10.1016/S1091-8531(97)90044-6 10532770

[32] Cheung PY Vickar DB Hallgren RA Finer NN Robertson CM. Carotid artery reconstruction in neonates receiving extracorporeal membrane oxygenation: a 4-year follow-up study. Western Canadian ECMO follow-up group. J Pediatr Surg. (1997) 32:560–4. 10.1016/S0022-3468(97)90707-7 9126754

[33] Henzler C Zöllner FG Weis M Zimmer F Schoenberg SO Zahn K. Cerebral Perfusion after repair of congenital diaphragmatic hernia with common carotid artery occlusion after ECMO therapy. In Vivo. (2017) 31:557–64. 10.21873/invivo.11094 28652420 PMC5566903

[34] Kurkluoglu M Badia S Peer SM Jonas R Shankar V Sinha P. Patency of common carotid artery and internal jugular vein after a simple vessel sparing cannulation for extracorporeal membrane oxygenation support. J Pediatr Surg. (2017) 52:1806–9. 10.1016/j.jpedsurg.2017.08.001 28916048

[35] Duggan EM Maitre N Zhai A Krishnamoorthi H Voskresensky I Hardison D. Neonatal carotid repair at ECMO decannulation: patency rates and early neurologic outcomes. J Pediatr Surg. (2015) 50:64–8. 10.1016/j.jpedsurg.2014.10.029 25598095 PMC5285515

[36] Levy MS Share JC Fauza DO Wilson JM. Fate of the reconstructed carotid artery after extracorporeal membrane oxygenation. J Pediatr Surg. (1995) 30:1046–9. 10.1016/0022-3468(95)90339-9 7472930

[37] Mendelson SJ Prabhakaran S. Diagnosis and management of transient ischemic attack and acute ischemic stroke: a review. JAMA. (2021) 325:1088–98. 10.1001/jama.2020.26867 33724327

[38] Winn HR Richardson AE Jane JA. Late morbidity and mortality of common carotid ligation for posterior communicating aneurysms a comparison to conservative treatment. J Neurosurg. (1977) 47:727–36. 10.3171/jns.1977.47.5.0727 908936

[39] Lago P Rebsamen S Clancy RR Pinto-Martin J Kessler A Zimmerman R. MRI, MRA, and neurodevelopmental outcome following neonatal ECMO. Pediatr Neurol. (1995) 12:294–304. 10.1016/0887-8994(95)00047-J 7546003

[40] Azapagasi E Kendirli T Tunçer GO Perk O Isikhan SY Tiras ST. Early neurologic complications and long-term neurologic outcomes of extracorporeal membrane oxygenation performed in children. Klin Padiatr. (2022) 234:96–104. 10.1055/a-1749-6096 35189653

[41] Bauer Huang SL Said AS Smyser CD Lin JC Guilliams KP Guerriero RM. Seizures are associated with brain injury in infants undergoing extracorporeal membrane oxygenation. J Child Neurol. (2021) 36:230–6. 10.1177/0883073820966917 33112194 PMC8086759

[42] Sansevere AJ DiBacco ML Akhondi-Asl A LaRovere K Loddenkemper T Rivkin MJ. EEG features of brain injury during extracorporeal membrane oxygenation in children. Neurology. (2020) 95:e1372–80. 10.1212/WNL.0000000000010188 32631921

[43] Trittenwein G Plenk S Mach E Mostafa G Boigner H Burda G. Quantitative electroencephalography values of neonates during and after venoarterial extracorporeal membrane oxygenation and permanent ligation of right common carotid artery. Artif Organs. (2006) 30:447–51. 10.1111/j.1525-1594.2006.00240.x 16734596

[44] Roelants-van Rijn AM van der Grond J de Vries LS Groenendaal F. Cerebral proton magnetic resonance spectroscopy of neonates after extracorporeal membrane oxygenation. Acta Paediatr. (2001) 90:1288–91. 10.1111/j.1651-2227.2001.tb01577.x 11808901

[45] Reitman AJ Chapman R Stein JE Paquette L Panigrahy A Nelson MD. The impact of venoarterial and venovenous extracorporeal membrane oxygenation on cerebral metabolism in the newborn brain. PLoS ONE. (2016) 11:e0168578. 10.1371/journal.pone.0168578 28033354 PMC5199081

[46] Reiterer F Resch E Haim M Maurer-Fellbaum U Riccabona M Zobel G. Neonatal extracorporeal membrane oxygenation due to respiratory failure: a single center experience over 28 years. Front Pediatr. (2018) 6:263. 10.3389/fped.2018.00263 30320047 PMC6167543

[47] Schiller RM Tibboel D. Neurocognitive outcome after treatment with(out) ECMO for neonatal critical respiratory or cardiac failure. Front Pediatr. (2019) 7:494. 10.3389/fped.2019.00494 31850291 PMC6902043

[48] Baumgart S Streletz LJ Needleman L Merton DA Wolfson PJ Desai SA. Right common carotid artery reconstruction after extracorporeal membrane oxygenation: vascular imaging, cerebral circulation, electroencephalographic, and neurodevelopmental correlates to recovery. J Pediatr. (1994) 125:295–304. 10.1016/S0022-3476(94)70214-4 8040781

[49] Papademetriou MD Tachtsidis I Elliot MJ Hoskote A Elwell CE. Multichannel near infrared spectroscopy indicates regional variations in cerebral autoregulation in infants supported on extracorporeal membrane oxygenation. J Biomed Opt. (2012) 17:067008. 10.1117/1.JBO.17.6.067008 22734786

[50] Tsou PY Garcia AV Yiu A Vaidya DM Bembea MM. Association of cerebral oximetry with outcomes after extracorporeal membrane oxygenation. Neurocrit Care. (2020) 33:429–37. 10.1007/s12028-019-00892-4 31925732 PMC7842183

[51] Joram N Beqiri E Pezzato S Andrea M Robba C Liet JM. Continuous monitoring of cerebral autoregulation in children supported by extracorporeal membrane oxygenation: a pilot study. Neurocrit Care. (2021) 34:935–45. 10.1007/s12028-020-01111-1 33029743

[52] Saito J Takekawa D Kawaguchi J Suganuma T Konno M Noguchi S. Preoperative cerebral and renal oxygen saturation and clinical outcomes in pediatric patients with congenital heart disease. J Clin Monit Comput. (2019) 33:1015–22. 10.1007/s10877-019-00260-9 30666542

[53] Chen S Fang F Liu W Liu C Xu F. Cerebral tissue regional oxygen saturation as a valuable monitoring parameter in pediatric patients undergoing extracorporeal membrane oxygenation. Front Pediatr. (2021) 9:669683. 10.3389/fped.2021.669683 34178887 PMC8220806

[54] Vedrenne-Cloquet M Lévy R Chareyre J Kossorotoff M Oualha M Renolleau S. Association of cerebral oxymetry with short-term outcome in critically ill children undergoing extracorporeal membrane oxygenation. Neurocrit Care. (2021) 35:409–17. 10.1007/s12028-020-01179-9 33432528

[55] McDevitt WM Farley M Martin-Lamb D Jones TJ Morris KP Seri S. Feasibility of non-invasive neuro-monitoring during extracorporeal membrane oxygenation in children. Perfusion. (2022) 1–10. 10.1177/02676591211066804 35212252

[56] Boyle K Felling R Yiu A Battarjee W Schwartz JM Salorio C. Neurologic outcomes after extracorporeal membrane oxygenation: a systematic review. Pediatr Crit Care Med. (2018) 19:760–6. 10.1097/PCC.0000000000001612 29894448 PMC6086744

[57] Lorusso R Taccone FS Belliato M Delnoij T Zanatta P Cvetkovic M. Brain monitoring in adult and pediatric ECMO patients: the importance of early and late assessments. Minerva Anestesiol. (2017) 83:1061–74. 10.23736/S0375-9393.17.11911-5 28643997

[58] Bembea MM Felling RJ Caprarola SD Ng DK Tekes A Boyle K. Neurologic Outcomes in a two-center cohort of neonatal and pediatric patients supported on extracorporeal membrane oxygenation. ASAIO J. (2020) 66:79–88. 10.1097/MAT.0000000000000933 30681441 PMC7765760

[59] Lin N Flibotte J Licht DJ. Neuromonitoring in the neonatal ECMO patient. Semin Perinatol. (2018) 42:111–21. 10.1053/j.semperi.2017.12.007 29397959 PMC5875727

[60] Ijsselstijn H Schiller RM Holder C Shappley RKH Wray J Hoskote A. Extracorporeal Life Support Organization (ELSO) guidelines for follow-up after neonatal and pediatric extracorporeal membrane oxygenation. ASAIO J. (2021) 67:955–63. 10.1097/MAT.0000000000001525 34324443

[61] Hendrikse J de Vries LS Groenendaal F. Magnetic resonance angiography of cerebral arteries after neonatal venoarterial and venovenous extracorporeal membrane oxygenation. Stroke. (2006) 37:e15–7. 10.1161/01.STR.0000198880.28827.84 16385098

[62] Lamers LJ Rowland DG Seguin JH Rosenberg EM Reber KM. The effect of common origin of the carotid arteries in neurologic outcome after neonatal ECMO. J Pediatr Surg. (2004) 39:532–6. 10.1016/j.jpedsurg.2003.12.005 15065022

[63] Takashima M Ray-Barruel G Ullman A Keogh S Rickard CM. Randomized controlled trials in central vascular access devices: a scoping review. PLoS ONE. (2017) 12:e0174164. 10.1371/journal.pone.0174164 28323880 PMC5360326

[64] Atay S Sen S Cukurlu D. Incidence of infiltration/extravasation in newborns using peripheral venous catheter and affecting factors. Rev Esc Enferm USP. (2018) 52:e03360. 10.1590/s1980-220x2017040103360 30304200

[65] Annich GM. Extracorporeal life support: the precarious balance of hemostasis. J Thromb Haemost. (2015) 13 Suppl 1:S336–42. 10.1111/jth.12963 26149045

[66] Van Ommen CH Neunert CE Chitlur MB. Neonatal ECMO. Front Med (Lausanne). (2018) 5:289. 10.3389/fmed.2018.00289 30410882 PMC6209668

[67] Kamdar A Rintoul N Raffini L. Anticoagulation in neonatal ECMO. Semin Perinatol. (2018) 42:122–8. 10.1053/j.semperi.2017.12.008 29336832

[68] Menaker J Tabatabai A Rector R Dolly K Kufera J Lee E. Incidence of cannula-associated deep vein thrombosis after veno-venous extracorporeal membrane oxygenation. ASAIO J. (2017) 63:588–91. 10.1097/MAT.0000000000000539 28857905

[69] Abruzzo A Gorantla V Thomas SE. Venous thromboembolic events in the setting of extracorporeal membrane oxygenation support in adults: a systematic review. Thromb Res. (2022) 212:58–71. 10.1016/j.thromres.2022.02.015 35219933

[70] Bidar F Lancelot A Lebreton G Pineton de. Chambrun M Schmidt M Hékimian G. Venous or arterial thromboses after venoarterial extracorporeal membrane oxygenation support: frequency and risk factors. J Heart Lung Transplant. (2021) 40:307–15. 10.1016/j.healun.2020.12.007 33422407

[71] Fisser C Reichenbächer C Müller T Schneckenpointner R Malfertheiner MV Philipp A. Incidence and risk factors for cannula-related venous thrombosis after venovenous extracorporeal membrane oxygenation in adult patients with acute respiratory failure. Crit Care Med. (2019) 47:e332–9. 10.1097/CCM.0000000000003650 30855325

